# ANCA Testing in Clinical Practice: From Implementation to Quality Control and Harmonization

**DOI:** 10.3389/fimmu.2021.656796

**Published:** 2021-03-16

**Authors:** Jan Damoiseaux

**Affiliations:** Central Diagnostic Laboratory, Maastricht University Medical Center, Maastricht, Netherlands

**Keywords:** vasculitis, proteinase 3, myeloperoxidase, standardization, harmonization

## Abstract

Analyses for the presence of anti-neutrophil cytoplasmic antibodies (ANCA) are important in the diagnostic work-up of patients with small vessel vasculitis. Since current immuno-assays are predominantly designed for diagnosis of patients with ANCA-associated vasculitis (AAV), implementation in routine clinical practice, internal and external quality control, and harmonization are focused on this particular use. However, ANCA testing may also be relevant for monitoring therapy efficacy and for predicting a clinical relapse in AAV patients, and even for diagnostic purposes in other clinical situations. In the current review, the topics of implementation, quality control, and standardization vs. harmonization are discussed while taking into account the different applications of the ANCA assays in the context of AAV.

## Introduction

The history of the detection of anti-neutrophil cytoplasmic antibodies (AAV), with hallmark developments, has been described before (1–3). With the exception of the European Medicines Agency (EMEA) classification algorithm for epidemiological studies (4), ANCA are not included yet in the classification criteria for the distinct entities of ANCA-associated vasculitis (AAV), i.e., (eosinophilic) granulomatosis with polyangiitis [(E)GPA] and microscopic polyangiitis (MPA), but it has been recommended for future criteria (5, 6). Moreover, ANCA are included in the Chapel Hill definitions of the vasculitides (7). Altogether, ANCA are well-recognized as a diagnostic biomarker, but the usefulness for follow-up remains a matter of discussion (8–11).

For diagnostic purposes, ANCA screening was originally performed by indirect immunofluorescence (IIF) assays on a substrate of ethanol-fixed neutrophils (12, 13). Positive samples were to be analyzed for reactivity to proteinase 3 (PR3) and myeloperoxidase (MPO) (14). Continuous improvement of the antigen-specific immunoassays has recently proven to be superior in performance as compared to IIF (15). This finding has precipitated in a revised consensus on ANCA testing for the diagnosis of AAV (3, 16). The new consensus states that high-quality immunoassays should be used as the primary screening method for patients suspected of having AAV, without the categorical need for IIF. A second immunoassay should be considered for negative results in patients with a high clinical suspicion (to increase sensitivity) or in case of low antibody levels (to increase specificity). There is no consensus published on how ANCA testing should be performed for monitoring AAV patients, but it seems obvious that the quantitative assay that revealed a positive result at the time of diagnosis is also to be used for follow-up.

Evidently, ANCA testing can be used in different clinical settings. For diagnostic purposes, routine screening may require different test characteristics than situations that demand a test result within 24 h (rapid ANCA test), like clinical manifestations associated with the renal-pulmonary syndrome. In the latter situation there is already a high pre-test probability for AAV (17) and simultaneous detection of anti-GBM antibodies is highly recommended (10). Also the use for screening vs. confirmation, or screening vs. follow-up, may have implications for choosing the most optimal assay. While for rapid testing and/or confirmation a qualitative result may be sufficient, quantitative results will improve the diagnostic value (*vide infra*) and are essential for follow-up.

This paper summarizes the distinct items to be taken into account for antigen-specific ANCA testing, i.e., MPO- and PR3-ANCA, in routine clinical practice with respect to implementation, quality control, and standardization. These items could be used in further discussions and, eventually, be implemented in recommendations and/or guidelines.

## Implementation of ANCA Assays in Clinical Practice

Since the ANCA test can be applied for different purposes, i.e., routine diagnosis, rapid diagnosis, confirmation, and follow-up, a combination of assays from different suppliers may be most optimal. However, from an health-economic perspective it makes sense to use assays for both MPO- and PR3-ANCA from the same supplier and to use these assays for both diagnostic as well as follow-up purposes. As such, it is most appropriate to use quantitative assays, while keeping in mind that the assays are primarily designed for diagnostic purposes. For diagnosis quantitative ANCA results are important because higher ANCA levels are associated with higher likelihood ratios and, therefore, with increased certainty of the right diagnosis (18, 19). For follow-up it is important to monitor possible decreases in ANCA levels upon therapy, but also to monitor possible increases as potential predictor for an upcoming relapse (10, 11). Obviously, for confirmation a distinct ANCA assay has to be used; also for rapid testing a distinct ANCA assay may be more suitable. Choosing the most suitable ANCA assay is the responsibility of the laboratory specialist, but should be discussed and communicated with the involved clinicians. The eventual choice will depend on the number of tests to be performed, the possibilities for automation, and financial resources, but also on local availability and/or approval by the authorities of the respective assay. Minimal requirements for the distinct applications of the ANCA assays is summarized in Table 1.

**Table 1:** Minimal requirements for implementation of ANCA assays in clinical practice.

**Requirement→ Clinical purpose↓**	**Automation^a^**	**Type of result^b^**	**Interpretation**	**Remark**
Routine screening	Yes	Quantitative	Likelihood ratio for test-result intervals	For follow-up end-point results to be determined
Confirmation	No	Qualitative	Single cut-off	Can be outsourced to reference laboratory
Rapid testing	No	Qualitative	Single cut-off	Anti-GBM antibodies to be included; for follow-up to be quantified in routine assay
Therapy follow-up	Yes	Quantitative	% relevant decrease	To be determined in similar dilutions
Prediction relapse	Yes	Quantitative	% relevant increase	To be determined in similar dilutions

a *Automation includes data exchange with the laboratory information system because this reduces administrative errors; for confirmation and rapid testing the numbers are expected to be rather low, making automation less advantageous*.

b *Optimally, all results are quantitative*.

Data on clinical evaluations of the diagnostic performance of distinct ANCA assays are widely available in the literature. It is a responsibility of the diagnostic industry to establish such studies in collaboration with relevant stakeholders. Authorative bodies, like the Food and Drug Administration (FDA), often require adequate study results before an assay is allowed to enter the market, but these data are most often not available to the community. In light of the *in vitro* diagnostics regulation (IVD-R; EU IVDR 2017/746) the sharing of study results will be an obligation for the diagnostic industry as of 2022 onward within the European Community (20). For clinical evaluation, however, it is important to keep in mind the intended use of the test and to evaluate the test accordingly for both MPO- and PR3-ANCA. For diagnostic purposes diagnostic samples, but not follow-up samples, and relevant disease controls are to be included. Analysis of a large cohort of apparently healthy controls, as required by the FDA, is of limited value for clinical practice, because the assays should not be used for population screenings. For rapid testing only samples from patients presenting with a pulmonary-renal syndrome, including rapidly progressive glomerulonephritis and/or alveolar hemorrhage, are relevant for analysis. Clinical evaluation of a confirmation assay is even more challenging because such evaluation depends on the choice of the screening assay; it is the algorithm that should be evaluated, not the overall diagnostic performance of the confirmation assay. For follow-up of AAV patients, the antigen-specific ANCA assay that was positive at the time of diagnosis is preferentially used; like for diagnostic approaches, the added value of simultaneously measuring an ANCA IIF titer is limited. It is important, however, to determine a clinically relevant decrease and/or increase and this is, among other items, dependent on inter- and intra-assay variability and, therefore, may differ for low, medium and high ANCA levels. In addition, it should be taken into account that quantification of ANCA levels may be hampered by the lack of linearity of many ANCA assays due to the heterogeneous nature of the measurant, i.e., the composition of low, medium and high affinity antibodies. If the measuring range of the assay is limited, one or more dilutions have to be analyzed to obtain a final quantitative result. Upon dilution the low affinity antibodies will increasingly take part in the equilibrium between free and antigen-bound antibodies and, as such, in the test result. Unfortunately, there is no consensus on the dilution steps to be used and the kit inserts do not give clear instructions on this issue, but it is evident that reliable interpretation of results in follow-up samples requires that the samples preferentially have been analyzed in the same dilution and in the same run. For prediction of relapses in AAV patients with PR3-ANCA a clinically relevant increase of 50–200% has been defined by receiver operating curve (ROC) characteristics for distinct ANCA assays (21–23). For patients with MPO-ANCA such data are not available.

Beside clinical evaluation, laboratory evaluation is an important step in the implementation of appropriate ANCA assays. This is the responsibility of the laboratory specialist and is dictated by accreditation bodies in documents like ISO 15189 (24). However, the requirements are primarily based on assays used in clinical chemistry and are ill-defined for autoantibody testing (25). Recently, a European hand-out on accreditation for laboratories involved in autoantibody testing has been formulated by the European Autoimmunity Standardization Initiative (EASI) (26). The hand-out is primarily focused on commercially available assays for clinical purposes. For in-house assays there exist detailed protocols (13, 27), but they require a more extended validation, which is beyond the scope of the current paper. Important items for the laboratory evaluation are reproducibility (intra- and inter-assay variability), carry-over in analyzers, and linearity (*vide supra*). Data on reproducibility of distinct methods for autoantibody detection, including ANCA, have been recently published (28). In this French EASI study, based on data obtained from French laboratories, the coefficient of variation (CV) is reported as the lowest CV value that is reached by 90% (CV90) and 50% (CV50) of the participating laboratories. The intra-run CV90 is about 10% for low, medium and high ANCA levels; the inter-run CV90 is about 15%. Similar results are reported for both MPO- and PR3-ANCA. Overall, chemiluminescent immuno-assays (CLIA) perform better than enzyme-linked immuno-sorbent assays (ELISA), but this may not apply for all CLIA and ELISA. Knowing the CV values of the assays is relevant in the diagnostic setting, in particular for test results close to the upper limit of normal. As a consequence, low ANCA levels have a relatively low likelihood ratio and, hence, require confirmation by an alternative assay (3). As already mentioned, CV values are also important for appropriate interpretation of changes in ANCA levels during follow up of patients with AAV.

Finally, in order to evaluate the clinical and laboratory performance of an ANCA assay to be implemented in clinical practice, sufficient samples with relevant clinical information should be available. For many laboratories this is a challenge because AAV is a relatively rare disease and for rare diseases it takes time to prospectively collect sufficient samples for the clinical purpose the assay is to be evaluated. Long-term storage capacity, therefore, is detrimental for clinical laboratories involved in autoantibody testing. Storage should not be restricted to positive samples, because negative samples of AAV patients are important to examine sensitivity, while the negative samples will most often represent relevant disease controls. A multi-center approach can facilitate acquisition of sufficient patient samples as effectuated for clinical evaluation (15), but can also be extended for the laboratory evaluation. Indeed, a Dutch initiative enables the laboratory evaluation according to ISO 15189 in a multi-center approach (29). Data obtained in the latter evaluation do not completely safeguard from a local evaluation, but this can be rather limited. If a laboratory even has insufficient samples available for such limited local evaluation, it should be questioned if the respective laboratory will maintain sufficient expertise in running the test and in interpreting the result. It is not the mere availability of an analyzer that should trigger the implementation of ANCA testing, but the more the expertise of the laboratory specialist involved in the interpretation of the results in the clinical context of the patient. The number of tests performed in a defined span of time to keep up sufficient expertise, however, has not been defined, but eventually may be addressed in accreditation processes.

## Quality Control

Since the results, both qualitative as well as quantitative, of ANCA tests are important in the diagnosis and follow-up of AAV patients, it is detrimental to monitor the quality of the reagents and assay performance. This demands for control at multiple levels, i.e., control of reagents at the time of arrival in the laboratory, internal quality control (IQC) and external quality control (EQC). Optimal quality control depends on the number of requests per time span. Laboratories with low numbers of requests not only will experience a problem with the laboratory evaluation at the time of implementation of the ANCA assay, but also will have an inefficient ratio between workload for patient care and quality control. Quality control guidelines were first formulated in the addendum to the 1999 international consensus statement on testing and reporting of ANCA (30). At that time clinical laboratories were more often using in-house assays, the IIF test on ethanol-fixed neutrophils still was the first choice for ANCA screening, and antigen-specific assays for detection of MPO- and PR3-ANCA were limited to ELISA. Nowadays, the revised consensus on ANCA testing prescribes to use antigen-specific assays for screening for which multiple distinct assay-types are available (3). Moreover, the initially formulated quality control guidelines are currently integrated in the documents for accreditation (24). Therefore, quality control of ANCA assays is not different from quality control of other autoantibody assays.

The quality of the reagents is primarily to be checked by the supplier of the assay upon production of a new lot of the respective reagent. However, the extent of this control can differ between diagnostic companies and the results are not extensively communicated upon distribution of the reagents. Therefore, it is mandatory to check the reagents of a specified lot before usage in clinical practice. This can be achieved by measuring a number of samples with a pre-defined target value. Evidently, this approach is based on the assumption that intra-lot quality is rather constant as it is impossible to test, for instance, all wells of an ELISA-plate. Multiplex assays, like addressable laser bead immunoassays (ALBIA) or line-immunoassays (LIA), on the other hand, may have an internal control in each single assay, but this, obviously, does not control for all reagents. Laboratories with a high number of ANCA requests may even be enabled to check several lot numbers before final acquisition, but this option is most often not available for laboratories with a relatively low number of requests. Besides errors in the production process, the quality of the reagents may also be affected during storage and subsequent transport to the clinical laboratory. This implies that not only subsequent lots have to undergo quality control upon arrival, but this also holds for separate deliveries of the same lot. Overall, the entry control of reagents, as prescribed for the ISO 15189 accreditation (24), benefits from ordering relatively large batches of the same lot, while keeping in mind the limited shelf-life of the reagents.

Most immunoassays contain a control to be used for IQC. If the result of this kit-control is within the limits as provided by the diagnostic company, the patient results obtained in the respective analysis can be approved and reported to the clinicians. It is questionable if a single kit-control is sufficient: the limits provided in the insert of the assay are rather broad, the kit-control most often is pre-diluted and stabilized, both resulting in a different matrix, and the control may not represent the complete analytical process. Furthermore, if the kit-control is integrated in a certain lot, it is not possible to identify lot-to-lot variation. Evidently, additional controls are mandatory in combination with more stringent acceptance rules. Besides, or possibly instead of, kit-controls, kit-independent controls, either derived commercially or home-made, should be included (30), preferentially to be used in the same dilution as patient material and by taking into account long-term stability. Replacement of the kit-control by an independent control, however, implies a modification of the assay and requires additional validation efforts according to the IVD-R (20, 26). In addition, distinct controls for multiple ANCA levels will enable to identify errors in different areas of the measuring range. In particular controls close to the cut-off or to the boundaries of test-result intervals may be of added value for IQC. Results of internal controls should be plotted serially into quality control charts and managed according to the Westgard rules by taking into account the CV values of the assay (28, 31). Actions to be undertaken upon aberrations should be pre-defined in the quality assurance documentation of the laboratory. Finally, before implementation of a new batch of control material a number of measurements is required to determine the target and CV value. In addition to IQC based on control samples, alternative data analyses enable to monitor the consistency in quality of ANCA assays. First, patient results can be retrospectively analyzed on the bases of percentage positive results within a predefined time-span. Depending on the chosen time-span and the number of ANCA requests this can be further fine-tuned for low-, medium-, and high-positive results. Changes over time may indicate a problem with the assay, but could also be due to, for instance, changes in requesting behavior or seasonal difference in relation to AAV. Another retrospective approach could be to randomly check if the final diagnosis is in line with the ANCA result, but for this approach one has to be aware that the ANCA results may be used to assign or reject the diagnosis of AAV. If aberrations are observed in such retrospective IQC, it is the responsibility of the laboratory specialist to inform the clinicians involved.

There exist multiple (inter)national organizations that facilitate EQC or proficiency testing. In some countries, there is a difference between EQC, which is performed on a voluntary basis, and proficiency testing, which is obligatory and involves restrictive measures (26). Participation in EQC, however, is mandatory for all parameters that are within the scope of ISO 15189 accreditation (20). Again, it is the responsibility of the laboratory specialist to choose an appropriate program reflecting the distinct ANCA assays offered in the clinical laboratory. There are substantial differences between the EQC programs with respect to how samples are selected and prepared, the number of samples that is being distributed, and the way the reported data are being analyzed. The primary objective of EQC programs is to evaluate if participating laboratories obtain the “right” results while using the standard procedures that are also used in routine clinical practice. This requires that EQC samples resemble patient samples. Since it is increasingly a challenge to obtain sufficient volumes of EQC samples, samples may be pooled, diluted, or derived from plasmapheresis material. This may introduce artifacts that become apparent in some assays, but not in others. However, such artifacts would never occur in a patient sample. Furthermore, in terms of autoantibody testing, the definition of a “right” result is difficult, in particular in defining a quantitative target value. Such target value might be defined by one or more reference laboratories, preferentially using different methods. However, often the consensus obtained by the participants is chosen as target value. In the latter case there is a bias toward the assay that is most prevalent in the participating laboratories. Since standardization is lacking in autoantibody assays (see next section), target values should be defined for each distinct assay and even cannot be generalized for, for instance, ELISA or CLIA. A second objective of an EQC program could be to increase awareness of differences between assays used in clinical practice. For instance, some assays for PR3-ANCA are more sensitive for ANCA present in patients with ulcerative colitis (32, 33). Such differences might be related to the cut-off chosen by the manufacturer or the way the autoantigen is processed. Knowledge of such advantages and/or limitations is important in the discussion with clinicians about possible discrepancies between the laboratory results and observed clinical manifestations (34, 35). Since there is an evident bias in the selection of samples for EQC (samples do not adequately represent the full spectrum of AAV patients), one should be very restricted in evaluating EQC data in terms of assay performance and testing algorithms (36, 37).

## Standardization vs. Harmonization

The perspective on standardization and harmonization of autoantibody assays has recently been extensively reviewed (35). The major conclusions are that standardization is a major challenge and has not yet been achieved, neither for ANCA assays, nor for autoantibody assays in general. Harmonization, on the other hand, may offer an alternative approach to better align requesting, testing, reporting and interpretation of autoimmune diagnostics.

Standardization is defined as “implementation of a standard preparation in order to maximize compatibility of test results, eventually resulting in uniformity of results”. For both MPO- and PR3-ANCA two distinct international standard preparations are available. First, the Autoantibody Standardizing Committee (ASC), a subcommittee of the International Union of Immunological Societies (IUIS) quality assessment and standardization committee has prepared standards for MPO- and PR3-ANCA (38). Both standards were assigned a value of 100 IU. Although several diagnostic companies have used these standards for calibration of their ANCA assays, this has not resulted in uniformity of results (39). Next, standards for MPO- and PR3-ANCA were prepared by the Institute for Reference Materials and Methods (IRMM), in collaboration with the Working Group Harmonization of Autoantibody Tests (WG-HAT) of the International Federation of Clinical Chemistry and Laboratory Medicine (IFCC) (40, 41). Although it was anticipated that these standards were better because of being commutable, the results were equally disappointing (42). The explanation for not achieving uniform results by the implementation of these standards, most likely is the heterogeneity of the measurant. Indeed, it can be anticipated that for each patient the composition of the autoantibodies will be different in terms of epitope recognition, affinity, isotype/subclass and glycosylation. This is elegantly illustrated for autoantibodies to dsDNA by Mummert et al. (43), and obviously also holds for MPO- and PR3-ANCA. Therefore, the source of the autoantigen, the way the autoantigen is presented in the immunoassay, and the composition of the conjugate are critical parameters for taking into account if standardization is to be achieved (35).

There is a split in the community between professionals that consider standardization achievable, vs. professionals that think standardization to be rather impossible. This issue is further complicated because the term standardization is often used where it actually involves harmonization, which is defined as “the adjustment of differences and/or inconsistencies among different measurements, methods, and procedures to make them uniform or mutually compatible.” In general this is achieved by consensus and is consolidated in recommendations and/or guidelines. For ANCA testing in the diagnostic setting harmonization starts at the requesting behavior (Table 2). For this purpose, both the 1999 and the 2017 international consensus on ANCA testing have defined the clinical manifestations associated with AAV that warrant an ANCA request (3, 14). Several studies have confirmed that this gating strategy results in a strong reduction of false-positive results without affecting the diagnosis of a true AAV patient (44–46). The second step in harmonization involves the type of test that is performed and the testing algorithm that is executed. According to the revised consensus, screening for ANCA is to be performed by high-quality assays for both MPO- as well as PR3-ANCA. Patients should be retested (preferentially with another antigen-specific solid-phase assay, or with IIF) only in case of a high clinical suspicion to increase sensitivity or a low-positive test result to increase specificity (3). IIF may be of added value in vasculitis cases for which other ANCA-specificities, like elastase-ANCA in drug-induced vasculitis, are suspected. Although the revised consensus originally only involved GPA and MPA, more recently consensus has been reached that for EGPA the same approach should be used (16). The third step in harmonization is the way test results are reported to the clinician. Traditionally, quantitative results are reported in combination with a single cut-off value that defines the result as negative or positive. Eventually, a gray-zone is introduced with a lower- and upper-limit of normal for which results are considered equivocal. As already mentioned, higher ANCA levels are associated with higher likelihood ratios and, therefore, with increased certainty of the right diagnosis. Indeed, the added value of a positive results for MPO- and PR3-ANCA improves with increasing levels of the autoantibodies (3, 15). Therefore, reporting results based on multiple cut-off values that identify negative, low positive, medium positive, and high positive results will further benefit the interpretation of the test result. With respect to harmonization, the multicenter study that was the basis of the revised consensus, interestingly, revealed that if results were reported in terms of likelihood ratios for test result intervals that were defined by pre-set levels of specificity, the likelihood ratios were very similar for the different assays included in the study (18). The level of harmonization that can be achieved by this approach is very promising and even resulted in a position paper, signed by relevant stakeholders in ANCA testing, that proposes to employ test result-specific likelihood ratios to align test result interpretation across assays and manufacturers and to convey clinical information intrinsic to the antibody level (19). Reporting test results as likelihood ratio will greatly facilitate interpretation of the results in the context of the clinical presentations of the patients, since there is a clear relationship, as defined by the Bayes theorem, between pre-test probability, likelihood ratio and post-test probability (47).

**Table 2:** Distinct levels of harmonization in ANCA testing for the diagnosis of AAV.

**Level of harmonization**	**Consensus and/or proposal**	**Responsibility**
Gating policy	Clinical manifestations for ANCA testing include: • Glomerulonephritis, especially rapidly progressive glomerulonephritis • Pulmonary hemorrhage, especially pulmonary renal syndrome • Cutaneous vasculitis with systemic features • Multiple lung nodules • Chronic destructive disease of the upper airways • Long-standing sinusitis or otitis • Subglottic tracheal stenoses • Mononeuritis multiplex or other peripheral neuropathy • Retro-orbital mass • Scleritis	Clinician
Testing algorithm	• Use high-quality immunoassays (both MPO- and PR3-ANCA) is recommended as the first screening method for detection of ANCA in patients suspected of AAV • If the result is negative and there is a high clinical suspicion of AAV, include a distinct antigen-specific immunoassay (or IIF) to increase sensitivity • If the result is low-positive, confirm the result with a distinct antigen-specific immunoassay (or IIF) to increase specificity	Laboratory specialist
Reporting of results	• Report quantitative results in combination with the cut-off value(s) provided by the manufacturer • If available, provide likelihood ratio's for test result intervals or communicate the test results associated with a likelihood ratio of 0.1, 1, 10 and 30 • In case of a rapid ANCA test, an initial qualitative result may be sufficient, but a note is to be added that the result of the routine quantitative ANCA test will follow	Laboratory specialist
Interpretation of results	• Interpret the result in the context of the clinical manifestations of the patient • Interpret the result in the context of the ANCA level • If available, interpret the likelihood ratio based on the Bayes theorem	Clinician

While reporting test results as likelihood ratios is a major step forward in harmonization of ANCA testing at the time of diagnosis, this does not apply for follow-up of patients with a definite diagnosis of AAV. Likelihood ratios defined for diagnosis are not to be confused with likelihood ratios required for showing efficacy of therapy or predicting a clinical relapse. Actually, likelihood ratios for follow-up of AAV patients are, if at all, only poorly defined. Hence, for follow-up there is no consensus with respect to ANCA testing. Due to the lack of standardization, however, it is evident that the same immunoassay is used, that quantitative results are reported and that the end-point level is measured while taking into account the non-linearity of most assays upon serum dilution. Finally, the report should define whether the change in ANCA level is relevant, for instance in respect to the risk of a clinical relapse as defined during the validation of the assay.

## Conclusions

Since the discovery that ANCA are associated with different entities of small vessel vasculitis, many improvements have been made in the overall quality of an ANCA result. This is due to technical improvements in the antigen-specific immunoassays, the regulations to be followed by both the diagnostic industry as well as the laboratories, but also the achievements made in terms of harmonization. It is evident that appropriate development, implementation and routine use of ANCA diagnostics requires collaboration between the diagnostic industry, laboratory specialists, clinicians, and, due to increasing ethical demands, also the patients and/or patient organizations. Interpretation of test results, in particular if reported as likelihood ratios, will be further facilitated if pre-test probabilities of distinct (combinations of) clinical manifestations are becoming readily available. Ideally, such information could be entered in the laboratory information system resulting in automatic calculation of the post-test probability based on the quantitative test result obtained. Currently, a large prospective multi-center study is ongoing that is intended to confirm the current international consensus on ANCA testing, but will also enable to strengthen the idea of harmonization by reporting in likelihood ratios. The reliability of the test result, obviously, is essential and requires implementation of high-quality ANCA assays and continuous monitoring of assay quality by IQC and EQC. In particular demands for appropriate IQC should be further defined by organizations involved in accreditation of clinical laboratories (25, 26). The use of assay-independent controls at 2–3 levels might become mandatory.

The improvements made are primarily focused on the added value of ANCA testing in the diagnosis of AAV patients. ANCA tests, however, are also used for follow-up of AAV patients and even beyond systemic vasculitis (48, 49). Obviously, for these situations there are specific demands that need to be further specified. In particular for follow-up ANCA testing in AAV patients in order to predict a clinical relapse there are multiple open issues: is it possible at all, for which patients this applies best (*cf*, MPO- vs. PR3-ANCA; limited vs. generalized AAV; primary small vessel vasculitis vs. drug-induced vasculitis), how is an ANCA-rise defined, which type of assay is to be used, do we need alternative IQC and EQC, and is harmonization feasible for this purpose. To answer these questions, well-designed, prospective multi-center studies are needed that also take into account novel immune-assays and therapeutic strategies, like B-cell depletion and complement inhibition. Unfortunately, there are no initiatives yet to organize such kind of study. For follow-up, currently, it is most important to use the same quantitative assay and to not confuse likelihood ratios defined for diagnostic purposes with those for predicting clinical outcome. Optimally, follow-up samples are analyzed together with the previous sample in the same dilution and the same run. Although this is evidently more expensive, it will provide a more accurate comparison that may prevent additional health-care costs and unnecessary stress in the patient. Hopefully, the next decade will enable to come to a consensus on ANCA testing beyond the diagnostic work-up of AAV patients.

## Author Contributions

JD was responsible for conception and writing of the whole manuscript.

## Conflict of Interest

JD has received lecture fees from several diagnostic companies, including Euroimmun, ThermoFisher, and Inova/Werfen.
